# Prosthetic Oral Rehabilitation with CAD/CAM Suprastructures in Patients with Severe Tissue Deficits: A Case Series

**DOI:** 10.3390/dj11120289

**Published:** 2023-12-13

**Authors:** Marisa Laurila, Pilvi Mäntynen, Jari Mauno, Juho Suojanen

**Affiliations:** 1 Päijät-Häme Joint Authority for Health and Wellbeing, Department of Oral and Maxillofacial Surgery, Lahti Central Hospital, 15850 Lahti, Finland; marisa.laurila@paijatha.fi (M.L.);; 2 Cleft Palate and Craniofacial Centre, Department of Plastic Surgery, Helsinki University Hospital, University of Helsinki, 00029 Helsinki, Finland; jari.mauno@hus.fi; 3 Clinicum, Faculty of Medicine, University of Helsinki, 00014 Helsinki, Finland

**Keywords:** prosthetic oral rehabilitation, CAD/CAM suprastructure, implant-supported, telescopic structure, fixed prosthodontics, oral cancer, facial trauma, syndrome, Atlantis, Createch

## Abstract

This article presents the outcomes of prosthetic oral rehabilitation using CAD/CAM telescopic bar overdentures in patients with oral cancer (*n* = 3), severe facial trauma (*n* = 2), or various syndromes (*n* = 1), all suffering from severe tissue deficits and requiring complex and comprehensive oral rehabilitation. The aim was to assess the durability and functionality of implant-retained prosthetic structures, ensuring easy oral hygiene and minimizing specialized follow-up needs. The data for this study were sourced from a retrospective cohort at Helsinki University Hospital. The prosthetic reconstruction encompassed the Atlantis 2in1 and the Createch removable telescopic systems. Thus, 40 implants were placed (4 to 7 per patient), with prosthetic structures in the maxilla (*n* = 4 patients), in the mandible (*n* = 1), and in both jaws (*n* = 1). Two patients experienced no complications, two patients had part of the acrylic resin break, and one patient experienced loosening of the bar structure. All complications associated with prosthetic structures were successfully managed, and none of the implants were lost. The follow-up time ranged from 7 to 126 months. This rehabilitation is proved to be an effective solution for patients with complex oral conditions, facilitating both functional restoration and ease of maintenance. These findings underscore the importance of individualized treatment approaches in cases of tissue deficits.

## 1. Introduction

Severe tissue deficits and complex oral conditions can result from oral cancer and its treatments, facial trauma, or various syndromes. In Finland in 2021, the incidence of pharyngeal and oral cancer (ICD10 codes C00-14) was 8.80 per 100,000 person-years (age-standardized to the Finnish population in 2014), totaling 283 cases [1]. As highlighted by Rogers in his review article, oral rehabilitation stands out as a primary concern affecting the quality of life for individuals after oral cancer treatment [2]. Moreover, individuals who experience severe facial trauma also endure significant and enduring health-related challenges, as well as challenges with life quality, due to these injuries. Nearly 4 out of 10 trauma patients with facial injuries (36%) experience functional limitations [3]. O’Connor et al. summarized in their review article that young males face a significantly elevated risk of facial injuries due to both interpersonal violence and sport activities. Substance abuse, including alcohol and drugs, contributes to a substantial portion of these injuries, accounting for 15–40% and 47% of injuries, respectively [4].

Oral cancer, as well as facial trauma and various syndromes, can lead to significant tissue deficits, including varying degrees of tooth loss. This often results in functional impairments and aesthetic concerns for the affected individuals. In the case of oral cancer patients, teeth with a poor prognosis are typically extracted before radiation therapy to prevent osteoradionecrosis. Protocols for tooth extraction in patients with head and neck cancer have been overlooked, and the need for extraction is determined more strictly by the prognosis and symptoms [5]. Although extractions may have been reduced, patients still frequently become edentate or partly edentate. Beumer et al. suggest that it is advisable to contemplate the extraction of mandibular molars exhibiting significant chronic periodontal bone loss and that are situated within the intended radiation zone before initiating the radiation treatment [6]. The rate of extraction varies, but in most clinics, teeth with a weak prognosis are extracted.

Prosthetic rehabilitation has proven to be essential for improving the quality of life for partly edentate and edentate patients [7,8]. When considering oral rehabilitation for edentulous jaws and evaluating the impact of immediate versus delayed loaded implants on oral-health-related quality of life (OHRQoL) and clinical outcomes, the overall scores did not reveal significant differences between the two groups. However, patients in the delayed loaded group displayed markedly higher levels of functional limitation and physical disability [9]. In cases of severe facial injuries, such as those caused by ballistic trauma, the focus is on providing sufficient bone material to facilitate dental implant placement. This approach is similar to the reconstruction methods used in oncology [10]. Implant-retained prostheses have emerged as a reliable solution for patients with severe tissue deficits. Ongoing advancements in implant dentistry, prosthetic technologies, and soft tissue reconstruction techniques offer promising avenues for improving the quality of life for these patients. For instance, in cases where patients with cleft lip and palate (CLP) experience extensive tissue deficits requiring intricate oral rehabilitation, utilizing a dental-implant-supported CAD/CAM bar with a removable telescope suprastructure provides a functional and easily maintainable solution for their rehabilitation requirements [11]. In cases involving complex oral conditions, removable dentures often prove to be an impractical treatment option. Patients undergoing oral cancer treatments, particularly those utilizing radiotherapy, which impacts the salivary glands and mucosa, experience facial challenges such as oral tissue dryness and reduced retention of removable dentures [12]. The use of removable dentures in oral cancer patients is associated with a decreased masticatory performance, especially in cases with a history of radiation therapy [13]. Moreover, individuals with head and neck cancer who also had undergone radiation therapy seemed to experience improved retention of prostheses in the mandible with implant overdentures (IODs) compared to complete dentures (CDs), and men may derive greater benefits of IODs compared to women [14]. Controversially, the impact of teeth and the type of denture appears to be relatively minor on the OHRQoL in patients with head and neck cancer. But patients with tumors located in the oral cavity had the highest mean score, signifying a poorer OHRQoL, in contrast to those with tumors in the nasopharynx, who exhibited the lowest values [15].

Dental implants provide an optimal solution for dental rehabilitation, offering improved prosthesis stability to individuals who have lost teeth due to tumors or treatment-related reasons. However, a patient’s eligibility for dental implant surgery depends on various factors, such as the risk of osteoradionecrosis after radiation therapy and meeting the necessary minimum bone height and width for successful implant placement, among other considerations [16]. Despite the potential benefits, the certainty regarding the success rate of implants in prosthetic-implant-based rehabilitation for patients with oral cancer remains elusive. Ettl et al. identified notable predictors for implant failure, including smoking, the use of bone grafts, and exposure to a radiation dose surpassing 60 Gy [17]. Additionally, Abdel Fattah et al. investigated the rehabilitation of patients with oral cancer following maxillectomy, emphasizing the importance of the pre-prosthetic surgical alterations in improving the prosthetic prognosis [18].

Oral rehabilitation of the patients suffering from severe tissue deficits constitutes a complex and evolving field that requires innovative solutions and multidisciplinary expertise. All patients in the cohort suffered from severe tissue deficits and required intricate and comprehensive oral rehabilitation. The primary aim of this study was to assess the durability and functionality of implant-retained prosthetic structures. This assessment aimed to promote the ease of oral hygiene maintenance and minimize the requirement for specialized follow-up care.

## 2. Materials and Methods

Patients in this case series were treated at the HUH Cleft Palate and Craniofacial Centre HUSUKE (Helsinki University Hospital, Helsinki, Finland). The data for this study were obtained from a retrospective cohort of patients (*n* = 6) suffering from major tissue deficits in the oral cavity due to cancer (*n* = 3), trauma (*n* = 2), or other syndromes (*n* = 1). The regional board of research accepted the research plan (ref: HUS/576/2019). The inclusion criteria were a CAD/CAM implant suprastructure having been built for the patient in the unit during the past decade, and the exclusion criteria were the diagnosis of a cleft lip and/or palate, as reported earlier. These patients required bone reconstruction and/or prosthetic oral rehabilitation to compensate for tissue deficits and improve oral function. The treatment options and associated risks were thoroughly evaluated and discussed with the patients. Although patients were not required to be completely smoking-free, they were instructed to limit smoking to a maximum of five cigarettes per day, and they were informed about the potential adverse effects of smoking on the success of the implant treatment. Implant surgery was performed under prophylactic antibiotics.

All patients received implant-retained removable prostheses, utilizing either the Atlantis 2in1 system (Dentsply Sirona, Charlotte, NC, USA) or the Createch removable telescopic system (Createch Medical S.L., Mendaro, Spain). Both systems are similar and consist of CAD/CAM primary and secondary suprastructures. The primary suprastructure is a milled titanium bar fixed to implants or implant bridge or bar abutments. The secondary suprastructure is removable and attaches to the primary suprastructure via friction with a milling degree of four degrees, along with additional retention elements. The secondary suprastructure contains a titanium framework covered with acrylic resin and custom teeth. The additional retention elements utilized in this case series included MK1 sliding bolt precision attachments (Mensadent, Plzeň, Czech Republic), CEKA attachments (Ceka Revax M2 Axial Titanax Bonding, Ceka Preci-Line, Alphadent NV, Waregeem, Belgium), or Locator attachments (Zest Dental Solutions, Carlsbad, CA, USA).

Most patients had already undergone comprehensive surgical treatment before their prosthetic treatment. Patients were clinically and radiologically examined by a prosthodontist before the stage of bony reconstruction. After a healing period of six months, the patients underwent radiological examination using cone beam computed tomography (CBCT) scans with tooth setups containing a radiological contrast agent. The final prosthetic plan was developed using CBCT data and implant planning software Romexis version 6.2.1.XX (Romexis, Planmeca, Helsinki, Finland). Following the completion of the treatment plan, patients underwent implant surgery, receiving four to seven implants according to the plan. The implants used were mainly Ankylos CX (Dentsply Sirona, Charlotte, NC, USA), and for one patient, Straumann WN (Straumann Holding AG, Basel, Switzerland) implants were used. After three to six months, when the implants were osseointegrated, the implants were uncovered, and the prosthetic phase of the treatment commenced.

Implant bridge abutments or bar abutments were installed for all patients at the level of the soft tissue margin. Primary impressions were then taken using polyether impression material (Impregum Penta Soft, 3M Oral Care, St. Paul, MN, USA), and the dental technician manufactured a chrome–cobalt (Cr–Co) frame and individual impression tray for the final precision impression. In the precision impression, the impression copings were splinted with the Cr–Co frame and autopolymerizing acrylic resin (Palavit G, Kulzer, Hanau, Germany) in order to achieve rigid splinting of the impression copings. For the precision impression, polyether impression material (Impregum Penta Soft, 3M Oral Care, St. Paul, MN, USA) was used, and if trimming was required, the material used was Kerr Impression Compound, green (Kerr, Uxbridge, Great Britain). Following the final impressions, the processed denture base with a wax occlusion rim was fitted and adjusted in the patients. During the last appointment, the denture base with a definitive tooth arrangement was evaluated and adjusted before being delivered with the definitive casts to the manufacturer of the primary and secondary suprastructures, either Dentsply Sirona or Createch, respectively. The casts and the denture base with the tooth arrangement were scanned and digitized by the manufacturer. The manufacturer was given information about the expected precision attachments and their amount and locations.

The suprastructures’ digital plans were evaluated by the prosthodontist before milling. If patients’ motor skills allowed, the most frequently used was the MK1 sliding bolts. Additionally, three or four Ceka or Locator attachments were used, but they were inactive in the secondary suprastructure when MK1 sliding bolts were used, and they were activated if necessary. However, the Ceka or Locator attachments were used and active in the nighttime splint, which was manufactured to guard the bar if necessary.

When the digital plans of the primary and secondary suprastructures were approved, they were milled and delivered to the dental technician. The suprastructures were fitted in patients, and the fit of the bar to the abutments was evaluated using the Sheffield test [19]. The tooth arrangement with the denture base was also evaluated with the CAD/CAM structures before the prosthetic structures were finalized by the dental technician.

The finalized prosthetic structures were placed in the patients. The primary bar suprastructure was anchored to the implant abutments using occlusal screws at specific torque settings. The secondary suprastructure was then evaluated on the bar, and necessary adjustments to the occlusion and acrylic resin contour were made. Patients also received occlusal splints designed at the same vertical height of the secondary suprastructure. Patients were instructed on how to properly use and remove the secondary suprastructure and how to clean the structures in order to maintain proper oral hygiene. Patients underwent check-up appointments at HUSUKE after one month, six months, and one year. After this period, they were referred to their dentist and dental hygienist for future care. In case any complications with the structures arose, patients were encouraged to contact HUSUKE or obtain a cover letter from their general dentist.

## 3. Results

**Figure 1:**
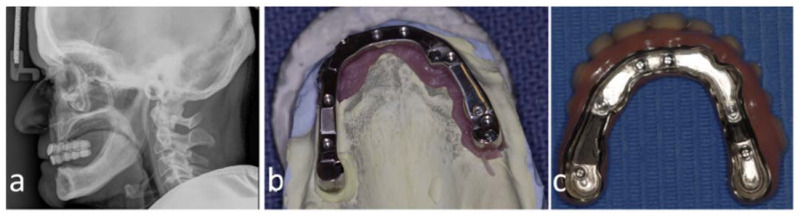
Preoperative cephalogram of patient no. 2 with barium-sulfate-dyed mockup prosthesis demonstrating the desired tooth positioning (**a**), manufactured maxillary bar by Createch (**b**), and a secondary suprastructure (**c**).

**Figure 2:**
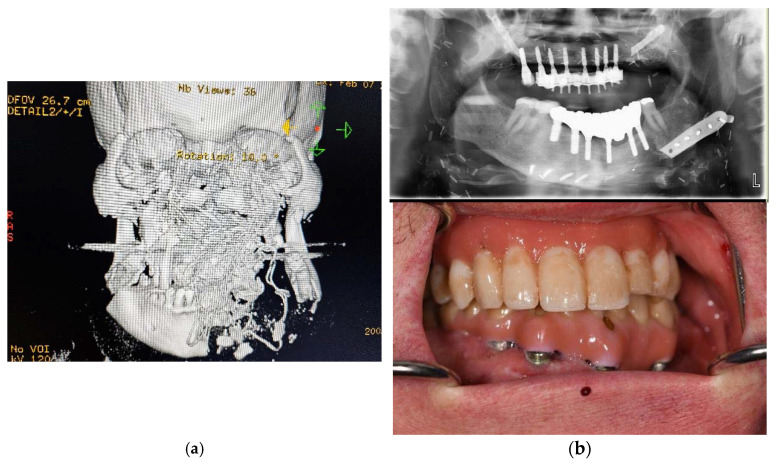
Primary computed tomography (CT) of patient no. 6 upon arrival (**a**). Most of the maxilla and nasal structures are missing, the outer frame of the zygomatic bones have comminuted fracture, and the mandible has comminuted fracture as well as some missing parts. The bleeding was tamponated with cloth, and the radio-opague marking of the tissue can be seen in the 3D reconstruction. The missing bone in the maxilla and mandible of the patient no. 6 had been reconstructed earlier with vascularized bone using microvascular free flap positioned in the desired vertical and horizontal position using virtual planning and CAM-produced osteosynthesis (Planmeca Promodel, Helsinki, Finland). The custom osteosynthesis from the reconstructed maxilla had been mostly removed for dental reconstruction (**upper b**). The final dental rehabilitation involved a two-in-one structure in the maxilla and a conventional implant bridge in the mandible (**lower b**).

This case series comprises a retrospective cohort of patients afflicted with severe tissue deficits in the oral cavity, necessitating prosthetic oral rehabilitation (Table 1). All patients were treated with implant-retained CAD/CAM removable prosthetic reconstruction, utilizing either the Atlantis 2in1 system or the Createch removable telescopic system (Figure 1 and Figure 2). Among these patients, three had battled carcinoma and undergone extensive disease treatment prior to prosthetic rehabilitation. Two others had experienced various facial traumas and had undergone surgical interventions before their prosthetic treatment. Additionally, one patient suffered from a syndrome leading to enamel hypomineralization, hypoplasia, and taurodontism [20], among other issues, and the patient was fully edentulous.

Five patients had undergone multiple surgical operations, and two patients had also received radiation therapy before prosthetic treatment (Table 2). Out of six patients, five received a full-arch prosthetic reconstruction, while one patient received a prosthetic structure consisting of nine units (Table 3). Prosthetic reconstruction was performed on the maxilla in four patients, on the mandible in one patient, and on both jaws in one patient. One patient underwent a sinus lift operation, and two patients underwent iliac crest augmentation procedures. Additionally, Bio-Oss (Geistlich Pharma AG, Wolhusen, Switzerland) was used in one of these latter-mentioned patients. Moreover, two patients had previously received vascularized bone grafts.

A total of 40 implants were placed in the patients, and none of the implants were lost. Three patients experienced complications with the prosthetic structures; two of these complications involved breaks in the acrylic teeth, occurring at 61 months and 81 months in one patient and at 38 months in another. These complications were successfully managed and repaired by the dental technician. In one patient, the bar became loose at 16 months, and the occlusal screws were replaced. The follow-up time varied from 7 months to 126 months.

## 4. Discussion

The aim of this study was to assess the durability and functionality of CAD/CAM implant-retained prosthetic structures in patients suffering from severe tissue deficits or fully edentulous jaws, requiring complex and comprehensive oral rehabilitation. The design of the rehabilitation focuses on ensuring the ease of oral hygiene maintenance and minimizing the requirement for specialized follow-up care, allowing it to be performed in local health centers.

Prosthetic rehabilitation for oral cancer patients poses significant challenges and requires a multidisciplinary approach [21]. Following surgery, patients may experience issues such as oro-nasal communication, speech difficulties, and eating challenges due to the resulting defects. Additionally, other challenges may arise, for example, those associated with the blood supply of a free flap when placing an implant during the rehabilitation process [22].

In their review article, Vosselman et al. emphasized the importance of the involvement of prosthodontists at the early stages of treatment for head and neck cancer patients [23]. Due to potential severe deficits resulting from head and neck cancer treatment, as observed in the patients in this case series, traditional removable prostheses may not be feasible for oral prosthetic rehabilitation [16]. Implant-retained prostheses have proven superior in terms of patient satisfaction among head and neck cancer patients compared to traditional prostheses [24]. Moreover, bar-retained implant overdentures were preferred by the head and neck cancer patients over Locator-type attachments in the overdentures in a retrospective study by Pieralli et al. [25]. Krennmair et al. compared different bar designs in their prospective study, and prosthetic structures with a round resilient bar demanded more prosthetic maintenance compared to rigid milled bar-retained overdentures [26]. In this case series, patients were provided with prosthetic reconstruction containing a rigid primary milled titanium bar fixed to the implant abutments and a secondary removable structure with a titanium framework covered with custom teeth and acrylic resin. This design aimed to provide a structure that was easy to clean, demanded less specialized maintenance care, and was as patient-friendly as possible given the patients’ backgrounds. The rigid structure of the prosthesis also reduces the pressure on the mucosa, which is an important consideration when planning prosthetic treatment for oral cancer patients who may have undergone radiation therapy and/or tissue reconstructions. Wolf et al. reported that, among other factors, the non-telescopic structure of the prosthesis had a negative impact on the implant success rates of head and neck cancer patients [27].

As mentioned earlier, Ettl et al. identified notable predictors for implant failure, including smoking, the use of bone grafts, and exposure to a radiation dose surpassing 60 Gy. Interestingly, in their study, the implants in the head and neck cancer patients exhibited an impressive overall survival rate of 92.3% after a two-year period, accompanied by a robust osseointegration rate of 94% and implant success rate of 78.6% [17]. Alberga et al. reported that in patients with head and neck cancer, implants should be inserted during the initial surgical procedure more frequently to minimize the need for additional surgeries, and they recommend this as a standard protocol at least in the native mandibular bone [12].

After orofacial trauma, patients may suffer from severe tissue deficits, and prosthetic rehabilitation is important to provide enhanced oral function and aesthetics. Before undergoing prosthetic treatment, patients may have already undergone surgical interventions depending on the severity of the trauma [28]. Various options such as removable dentures, implant-supported fixed prostheses, and implant-retained removable prostheses have been proposed and utilized for the prosthetic rehabilitation of dental trauma patients [28,29,30,31]. Brauner et al. preferred fixed implant-supported hybrid prostheses for patients with ballistic trauma to compensate for both hard and soft tissue deficits, and to offer easily cleanable prosthetic structures [28]. Tuna et al. reported on oral rehabilitation in a traumatic injury case where the patient declined bone augmentation and other surgical procedures. As an alternative, they applied a modified combination prosthesis with tissue ceramic and a zirconia-based crown, despite an implant-retained fixed prostheses being the first alternative [32]. Awadalkreem et al. presented a case series where patients with severe dentoalveolar trauma were treated using corticobasal implants without the need for bone grafts. The prostheses were fabricated using conventional techniques [33]. This, however, excludes severe tissue deficits.

There are alternative methods for rehabilitating complex oral conditions using CAD/CAM solutions compared to the CAD/CAM implant-supported telescopic bar dentures studied in this case series. Rahfl et al. utilized the IPS preprosthetic^®^ implant (KLS-Martin, Tuttlingen, Germany) in their study, focusing on oral rehabilitation for patients with cleft lips and palates facing challenging conditions in both the hard and soft tissues. Initially, this implant system was designed for post-ablation oncological patients and individuals with severely atrophic jaws. The study showcased the effective utilization of a one-piece multi-vector screw and its role in retaining a stable patient-specific implant (PSI) for prosthodontic rehabilitation in CLP patients, especially in those with deformities and difficult initial conditions. The advantages of this protocol lie in its approach, involving a single-step surgical procedure that allows for the immediate placement of a temporary denture. This approach offers the possibility of full primary loading, which stands in stark contrast to standard protocols that typically entail many stages, including bone grafts, dental implants, their uncoverage, and possibly free mucosal grafting [34]. However, this approach demands larger surgical openings under general anesthesia. The framework requires space between the malar bone and the zygomatic arch, so the anterior insertion of the masseter muscle must be dissected. Additionally, this approach necessitates more studies and long-term follow-up, for example, to deduce the complication rates.

Recently, more accessible use of CAD/CAM patient-specific solutions has made it possible to create larger bone-borne structures where bony defects are not reconstructed as a whole. Instead, the implant receives support from the existing facial bones and a large number of mini-screws, and dental reconstruction is built on top. Korn et al. demonstrated that following ablative tumor surgery, reconstruction can be achieved using patient-specific maxillary implants without the need for bone grafting [22]. Subperiosteal mini screw CAD/CAM structures have not been widely utilized, and there have been relatively few papers published on this matter.

In another study from the same group, dental rehabilitation of highly atrophic maxillas was carried out using subperiosteally placed and rigid multi-vector bone-anchored PSIs when conventional implants could not be placed due to a lack of bone. This approach resulted in clinically stable implants for prosthetic reconstructions, despite a few complications such as infection, the exposure of the framework, and screw fractures [35]. Jehn et al. also utilized patient-specific implants in their study. Patients were provided either with a fixed dental prosthesis or removable dentures supported by patient-specific implants; the OHRQoL of these patients was evaluated, and patient-specific implants especially with a fixed prosthesis proved to enhance the OHRQoL [36]. The use of bone- and mini-screw-borne custom structures is still quite limited, however, and susceptibility to infections with more regular use remains to be seen. One challenge for these patient-specific solutions is that the dental suprastructure often uses connections and screws not commonly used in average dental offices. This leads to a problem: in the case of denture failure, corrective treatment cannot be provided by regular dentists and dental technicians, and the follow-up must be carried out in specialized units. However, some manufactures, such as Createch and KLS Martin, have started collaborating with conventional dental implant manufacturers, such as Straumann, which makes this problem less evident and presumably lowers the barrier for use. In the future, conventional surgical osteosynthesis and surgical implant material providers will likely face increasing pressure to offer patient-specific solutions for oral and facial reconstruction.

An alternative technique for treating patients with severe atrophy in the maxilla is the placement of zygomatic implants. D’Agostino et al. reported an overall survival rate of 97.41%; complications for patients were sinusitis, zygomatic bone fracture, and oro-antral communication [37]. The challenge associated with zygomatic implants lies in the demanding insertion technique and their limitation in providing support only around the premolar or molar areas. Additionally, the position of the implant connector is often palatal, leading to compromises in the suprastructure design [38]. In their comprehensive review article, Brown et al. discussed various reconstructive approaches for the maxilla and midface in patients who have undergone surgical removal of malignant tumors. They discussed the potential use of obturator prostheses for smaller defects and highlighted the consensus on the necessity of free flaps in extensive maxillary reconstructions. Additionally, they emphasized the significance of composite flaps comprising both bone and muscle for enabling implant-borne dental prostheses. Furthermore, the authors pointed out the use of zygomatic implants to retain dentures in larger defects, though further longitudinal research is needed on this technique [39]. In their study, Morena et al. examined the clarity of speech and dietary changes after surgery in patients with moderate-sized maxillectomy defects, comparing those treated with obturators to those who underwent free flap procedures. Their comparison revealed that successful treatment of the palate is achievable using both methods. Moreover, in cases involving substantial palatal defects exceeding 50%, free flap reconstruction demonstrated superiority in both aspects [40].

In this case series, an analogical impression technique was utilized by the prosthodontist, and the definitive working model as well as the denture base with acrylic resin teeth were digitized using an industrial-level scanner by the manufacturer of the titanium CAD/CAM suprastructures. In the field of dentistry, the digital workflow is increasing and offering viable impression, design, and manufacturing protocols for prosthetic dentistry [41]. However, multiple different scanners and types of software are available, and features such as trueness, accuracy, and precision are important to comprehend and to be evaluated in order to obtain the proper information and possible limitations about the system used [42].

The follow-up periods vary, ranging from 7 to 126 months, and the data remain insufficient to conclusively determine long-term success rates. However, our unit is a tertiary center for severe facial and dental reconstructions, where the treatment is funded by the community, and the patients receive treatment with minor fees during appointments. The follow-up is normally arranged in local health centers, and the patients would most likely have been re-admitted to our center if problems had occurred with the prostheses. Unfortunately, the current legislation does not provide the possibility of contacting local health service providers outside the unit for further detailed inquiry for research purposes, so this remains at the level of speculation. Moreover, this study’s retrospective design, coupled with the small patient cohort, poses inherent limitations. Additionally, alterations to the patient health record system have made previously scanned anamnestic information inaccessible and unreliable for retrospective analysis. The evident lacking of this study includes a highly heterogenous and very small number of patients, as well as the heterogeneity in the prosthetic design of the suprastructures. Consequently, further research is imperative to provide comprehensive insights into the long-term survival and success rates of the prosthetic structures with a more specified patient cohort and prosthetic design.

## 5. Conclusions

Oral rehabilitation in patients with severe tissue deficits requires a multidisciplinary approach, and it is a complex and evolving field that demands innovative solutions and expertise. Advances in implant dentistry and prosthetic technologies are promising. Prosthetic oral rehabilitation employing CAD/CAM telescopic bar overdentures proved to be an effective solution for patients with complex oral conditions, facilitating both functional restoration and ease of maintenance, but further research especially on the long-term survival for this prosthetic structure is needed. These study findings underscore the importance of individualized treatment approaches in cases of tissue deficits, with favorable long-term outcomes observed in this diverse patient cohort.

## Figures and Tables

**Table 1:** Patient demographics.

ID	Gender	Diagnosis	Age	Opposing Dentition
1	F	Facial trauma	58	Own teeth
2	M	Tricho–dento–osseus syndrome	66	Paired two-in-one structure
3	M	Tonsillary carcinoma and cervical node metasthasis	55	Own teeth
4	F	Maxillary sinus carcinoma	82	Overdenture
5	M	Carcinoma of the floor of the mouth and tongue	66	Overdenture
6	M	Facial gunshot trauma	60	Own molars and fixed implant prosthesis region 35–45

Abbreviations: F, female; M, male.

**Table 2:** Surgical interventions and radiation therapy.

ID	Sinus Lift	Crest Augmentation	Previous Surgical Operations	Radiation Therapy
1	None	Iliac crest graft	Caldwell–Luc with l.a. Multiple periodontal operations. Free iliac bone grafts in premaxilla.	None
2	Bio-Oss, left	Bio-Oss, iliac crest	Extraction of all teeth	None
3	None	None	Tonsillectomy and resection of the base of the tongue, neck dissection, radial forearm flap reconstruction, and failure. Pectoralis major flap reconstruction. Extraction of decayed teeth.	60/2 Gy postoperatively
4	None	Vascularized bone	Partial maxillectomy. Radial bone-free graft reconstruction. Neck dissection with l.a. Removal of decayed teeth perioperatively. Fistula and non-union of free bone graft. Re-bone grafting with iliac crest chips. Facial artery muco-musculous rotation flap.	None
5	None	None	Resection of oral base and subtotal glossectomy, partial mandibulectomy, cervical lymph node dissection with l.a. Reconstruction with vertical rectus abdominis free flap.	60/2 Gy postoperatively
6	None	Vascularized bone	Fibula reconstruction in maxilla, microvascular reconstruction, trabecular bone and Bio-Oss in mandible, nasal radial forearm artery free flap reconstruction.	None

**Table 3:** Characteristic of patients.

ID	Number of Implants	Type of Implant	Type of Prosthetic Structure	Complications	Follow-Up (mo)
1	6 mx	Straumann WN	Createch, 9 units	None	7
2	7 mx, 6 mn	Ankylos CX	Createch, full arch maxilla and mandible	Acrylic teeth break at 61 mo and 81 mo	126
3	4 mx	Ankylos CX Ankylos CX	Atlantis 2in1	Loosening of bar structure at 16 mo	62
4	3 mx right, 3 free vascularized bone graft	Ankylos CX	Atlantis 2in1	None	14
5	4 mn	Ankylos CX	Atlantis 2in1	None	80
6	7 mx	Ankylos CX	Atlantis 2 in 1	Acrylic teeth break at 38 mo	39

Abbreviations: mx, maxilla; mn, mandible; mo, months.

## Data Availability

Single-patient data are not available due to the GDPR.
